# Metabolic Responses, Uptake, and Export of Copper in Cyanobacteria

**DOI:** 10.3390/biology14070798

**Published:** 2025-07-01

**Authors:** Jean Coutinho Oder, Thamires Emidio Sateles, Laila Barros de Souza, Adriano Nunes-Nesi, Wagner L. Araújo, Luna Alvarenga-Lucius

**Affiliations:** 1 Departamento de Biologia Vegetal, Universidade Federal de Viçosa, Viçosa 36570-900, Minas Gerais, Brazil; jean.oder@ufv.br (J.C.O.); thamires.sateles@ufv.br (T.E.S.); laila.b.souza@ufv.br (L.B.d.S.); nunesnesi@ufv.br (A.N.-N.); wlaraujo@ufv.br (W.L.A.); 2 Institute of Biosciences, Department of Plant Physiology, University of Rostock, D-18059 Rostock, Germany

**Keywords:** copper homeostasis, cyanobacterial stress response, copper toxicity

## Abstract

Copper (Cu) is an essential micronutrient for cyanobacteria, where it functions as a cofactor in key proteins involved in photosynthesis and antioxidant defense. However, at elevated concentrations, Cu becomes toxic, exhibiting algicidal effects by disrupting metal homeostasis and competing for metal-binding sites on critical cellular proteins. Due to the considerable morphological and physiological diversity within the phylum Cyanobacteria, the thresholds for Cu deficiency or toxicity vary considerably among strains. Maintaining Cu homeostasis in cyanobacterial cells is a complex process involving multiple layers of regulation. It begins at the extracellular polysaccharide layer, involves specialized membrane-bound proteins (in the outer, plasma, and thylakoid membranes), and results in transcriptional regulation in response to intracellular Cu status. This review summarizes the current understanding of Cu uptake and efflux pathways in cyanobacteria and explores how these mechanisms contribute to maintaining cellular Cu balance. The knowledge gained may contribute to the application of cyanobacteria in bioremediation strategies and/or the targeted use of Cu in the control of harmful cyanobacterial blooms.

## Simple Summary

Copper (Cu) is an essential micronutrient for cyanobacteria, participating in important cellular processes such as photosynthesis. However, at elevated concentrations, Cu becomes toxic to these organisms. This dual nature has led to the hypothesis that Cu could serve as an effective algaecide for controlling cyanobacterial blooms in aquatic environments. Cyanobacteria possess various cellular mechanisms for Cu uptake, homeostasis, and detoxification. Despite recent research efforts, many of these mechanisms remain incompletely understood, and the available results are fragmented across the literature. This review aims to compile and analyze current knowledge on the Cu uptake required for cellular function and the detoxification responses to excess environmental copper, particularly focusing on Cu extrusion mechanisms. These insights may contribute to the development of Cu-based strategies for the management of cyanobacteria blooms, as well as the use of cyanobacteria bioremediation of Cu-contaminated environments.

## 1. Introduction

Cyanobacteria, formerly known as blue-green algae, are a monophyletic group of oxygenic photosynthetic prokaryotes [1]. They exhibit extensive physiological and morphophysiological diversity, which enables them to colonize nearly all photic environments on Earth [2]. Due to their metabolic versatility, cyanobacteria have gained attention for their potential in various biotechnological and industrial applications, including the production of biodiesel and biofertilizers [3,4,5,6,7,8,9]. Cyanobacterial strains also exhibit variable yet consistent bioabsorption and bioaccumulation capacities for a range of toxic metals, including copper (Cu), nickel (Ni), cadmium (Cd), lead (Pb), mercury (Hg), zinc (Zn), chromium (Cr), and manganese (Mn) [10]. These traits make them promising candidates for targeted bioremediation and the removal of organic and inorganic contaminants from wastewater [7,10,11,12]. Furthermore, the potential to repurpose cyanobacterial biomass for industrial applications creates new opportunities for integrated waste treatment in effluents from sugarcane processing [13], dairy industries [14], rice [15], maize [16], paper [17], and textiles [8].

Cu is an essential micronutrient for cyanobacteria, playing a critical role in fundamental metabolic pathways. It acts as a cofactor in several enzymes involved in redox reactions, notably in photosynthesis, where it is crucial for the proper function of plastocyanin (PC) [18]. In the respiratory chain, Cu is a critical component of cytochrome c oxidase enzyme, which catalyzes the reduction of oxygen to water [19]. In addition, Cu contributes to the oxidative stress response through its association with Cu/Zn-superoxide dismutase (Cu/Zn-SOD), which catalyzes the dismutation of superoxide radicals into oxygen and hydrogen peroxide [20]. However, despite its biological importance, at elevated concentrations, Cu can exert toxic effects on cyanobacteria [21]. Anthropogenic activities, including mining and paint manufacturing, have significantly increased Cu levels in aquatic environments [22]. At elevated concentrations, Cu competes with other ions, particularly iron, for binding sites within the context of Fe-S containing enzymes. This can result in metabolic imbalances and subsequent cellular stress [11].

Here, we explored the molecular and physiological mechanisms underlying Cu uptake and response in cyanobacteria, with an emphasis on their potential contributions and applications in environmental remediation strategies.

## 2. Cu Essentiality, Deficiency, and Toxicity

Given its classification as a micronutrient, minimal quantities of Cu are required for its supply to cyanobacteria. Due to their important role in redox reactions, the absence of Cu can result in deficiencies in processes such as photosynthesis and cellular respiration in cyanobacterial cells. Furthermore, there is the possibility that it will influence environmental responses due to the disruption of the homeostasis of this element.

To date, the scarcity of research in this area is evident, with the majority of studies conducted on *Synechocystis* sp. PCC 6803. For example, it was observed that in the absence of Cu, the strain reduced the expression of PC, which contains Cu ions coordinated by amino acid residues, and increased the expression of cytochrome c6 (Cyt c6), which contains iron [23]. This substitution enables the cell to sustain electron transport under copper-deficient conditions by utilizing cytochrome c6 as an alternative electron donor to photosystem I (PSI), thereby supporting growth rates comparable to those observed under copper-sufficient conditions. Researchers further explored the regulation of the PC/Cyt c6 switch in the absence of Cu in *Synechocystis* sp. PCC 6803, which is regulated by the BlaI/CopY-family transcription factor PetR and the BlaR-membrane protease PetP [24]. PetP is a Cu-sensing membrane protease that regulates PetR. In the presence of Cu, PetP degrades PetR, thereby enabling elevated expression of PC. Conversely, in the absence of Cu, PetP becomes inactive and can no longer degrade PetR. Thus, PetR represses the expression of *petE* (the gene encoding PC) under limiting Cu, while simultaneously activating the transcription of *petJ* (the gene encoding Cyt c6) [24]. By varying its concentration, copper can be used in metal-regulated promoters responsive to this element to induce or repress the expression of specific genes, making it a highly functional tool for research routines. In addition to the “pet” system in cyanobacteria, copper-inducible genes have also been described in fungi and *Escherichia coli* [24,25,26]. While metal-regulated promoters are theoretically effective, practical applications often encounter challenges due to unintended basal expression. Even in media lacking added copper, trace amounts of copper can be present from residuals on glassware or in filtered water, leading to partial activation of the *petE* promoter. This phenomenon has been documented in various studies, highlighting the difficulty in achieving tight control over gene expression using this system. To address these challenges, researchers have employed strategies such as acid-washing glassware to eliminate residual copper and incorporating chelators, such as bathocuproinedisulfonic acid disodium salt, to sequester trace amounts of copper in the growth medium. In summary, while the *petE* promoter offers a copper-inducible mechanism for gene expression in cyanobacteria, careful consideration of experimental conditions and the implementation of appropriate control measures are essential to minimize unintended basal expression and ensure precise regulation [27].

The absence of literature on suboptimal doses of Cu indicates a potential area for further exploration in the cyanobacterial field. Moreover, the copper concentrations employed in various culture media may offer valuable insights into optimizing the use of suboptimal levels for growth and metabolic activity. The BG-11 medium, the most well-known and commonly used medium for culturing freshwater, non-nitrogen-fixing cyanobacteria, contains 0.316 μM of CuSO_4_·5H_2_O [28]. The ASN-III medium, utilized to replicate the marine environment for cyanobacteria from such environments, also contains 0.316 μM of CuSO_4_·5H_2_O [29]. The Z8 medium, which does not contain NaCl and is ideal for cyanobacteria that do not tolerate salinity, contains 5.01 µM of CuSO_4_·5H_2_O [30]. Finally, the Zarrouk medium, an alkaline medium used as a standard for *Arthrospira platensis* to promote growth and maximum biomass gain, also contains 0.316 μM of CuSO_4_·5H_2_O [31].

In the strain *Leptolyngbya* sp. GUEco1015, the capacity for Cu bioaccumulation and documented the consequences of elevated Cu doses [21]. The LC_50_ of Cu^2+^ for this strain was determined to be 14.17 μM (0.9 ppm). Excessive doses of 23.6 µM (1.5 ppm) led to severe chlorosis, filament disorganization, and a twofold increase in cellular H_2_O_2_ and MDA concentrations when compared to control (standard BG-11) [21]. Moreover, the effects of a 10 µM Cu^2+^ concentration on the cyanobacterial strain *Nostoc muscorum* was investigated with similar outcomes, including chlorosis and filament disorganization. This treatment led to an approximate 38% reduction in chlorophyll content, a 20% decrease in total protein content, and a 40% decline in photosynthetic potential [32]. A decrease in total protein content and an increase in free amino acids were also observed in *Spirulina platensis*. In a separate study, it was found that exposure to 7.87 µM, 15.74 µM, and 47.21 µM (0.5, 1.0, and 3.0 mg) of Cu^2+^ resulted in a continuous decline in total protein content (from 493.63 mg/L in the control to 182.47 mg/L at 3.0 mg) and a steady increase in free amino acids (~12.5 mg/L in the control to ~30 mg/L at 3.0 mg) [33]. Finally, in the model strain *Synechocystis* sp. PCC 6803, a 3 µM Cu^2+^ concentration was able to induce a fivefold increase in reactive oxygen species (ROS) production [34].

Advancing technologies for managing harmful cyanobacterial blooms require a detailed understanding of copper’s species-specific effects. Microcystis aeruginosa morphology significantly influences copper tolerance: unicellular strains exhibited over 95% loss in viability after 24 h of exposure to 3.93 µM (0.25 mg/L) Cu^2+^, while colonial strains retained higher viability under all conditions [35]. Previous findings on *M. aeruginosa* indicate that unicellular strains are significantly more sensitive to copper than many microalgae [36], reinforcing the potential of copper as a targeted control agent for this harmful species. However, copper-based compounds such as copper sulfate can induce the release of intracellular cyanotoxins, particularly microcystins, into the water column during cell lysis. Although effective in reducing biomass, this treatment does not eliminate toxin risk and may exacerbate water quality concerns [37]. Consequently, integrated management strategies are needed—ones that both control blooms and mitigate toxin release. Alternatives to copper include biological controls (e.g., algicidal bacteria, zooplankton grazers) [38], nutrient reduction (particularly phosphorus and nitrogen), and physical interventions (e.g., ultrasonic treatment, artificial mixing). For toxin removal, effective methods include activated carbon adsorption [39], advanced oxidation processes (e.g., ozonation, UV/H_2_O_2_) [40,41], and membrane filtration (e.g., nanofiltration, reverse osmosis) [42]. Combining these approaches provides a more sustainable and environmentally responsible framework for cyanobacterial bloom management.

In summary, while the toxic effects of Cu on cyanobacterial strains are broadly similar, the concentration threshold that elicits these effects varies considerably. This variability reflects the substantial morphological and metabolic diversity within the cyanobacterial phylum, making it likely that different strains exhibit distinct responses to equivalent Cu concentration. As such, determining a specific LD_50_ for each strain may be required to accurately characterize their sensitivity to Cu toxicity. Importantly, this review highlights an apparent trend of increased Cu sensitivity among unicellular strains compared to filamentous ones. Evidence suggests that Cu concentrations up to 10 µM can induce pronounced metabolic disruptions in unicellular cyanobacteria, whereas filamentous strains typically require higher concentrations to exhibit comparable physiological impairments.

## 3. Cu Transport: Extracellular to Intracellular Space

In *Nostoc calcicola*, it was observed that the absorption process of Cu^2+^ occurs in two phases: (1) a rapid binding of ions to the cell wall through charge interactions (around 10 min) and (2) the slower entry of ions into the intracellular environment (40 min to 1 h) [43]. The uptake and transport of ions across membranes are enhanced in photosynthetically active cells once they are exposed to light, as it has been proven that the ATP responsible for Cu transport is derived from PSII reactions [43].

Porins are well known for facilitating the passive transport of Cu^2+^ ions from the extracellular environment into the periplasmic space in bacteria [44]. The outer membrane of *Synechocystis* sp. PCC 6803 contains several porin-like proteins—such as Slr1841, Slr1908, and Slr0042—that are primarily involved in iron transport but also exhibit high permeability to other inorganic ions. This suggests that ions such as Cu^2+^ may gain rapid access to the periplasm via these protein channels [45]. These porins possess an N-terminal S-layer homology (SLH) domain, which anchors them to the polysaccharide component of the peptidoglycan layer, and a C-terminal transmembrane domain predicted to form a β-barrel structure [45,46]. The OprB domain of bacterial porins was originally identified as a sugar-specific domain involved in the recognition and transport of carbohydrates [47]. However, in *Anabaena* sp. PCC 7120, seven mutants lacking different OprB-domain-containing porins exhibited increased resistance to elevated copper concentrations, suggesting that many of these putative porins participate in copper transport [48]. Additionally, the OprB domain has also been shown to mediate the selective uptake of carbohydrates from the extracellular environment into the periplasm in cyanobacteria [49,50].

**Figure 1:**
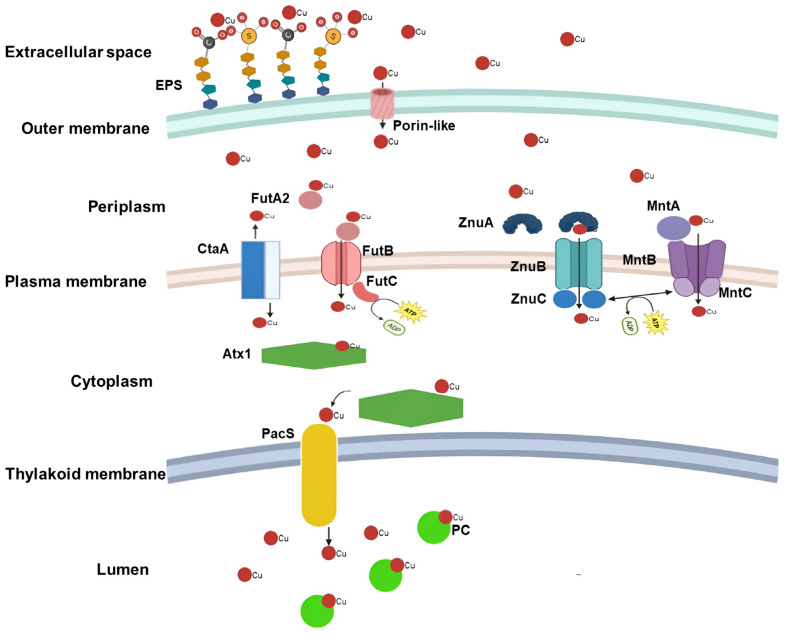
**Schematic representation of the passage of Cu ions from the extracellular environment into the cyanobacterial cell.** Interaction of Cu ions with negative charges present in the EPS layer. Entry by diffusion through porin-like structures into the periplasm and via the CtaA and FutABC (ATP-Binding Cassette transporter—iron ABC) proteins into the cytoplasm. Alternatively, passage into the cytoplasm by competing with other metal import systems, such as Zn (ZnABC) and Mn (MntABC)—ATP-Binding Cassette transporters—ABC. Arrows represent the Cu flux direction.

Copper may also enter cyanobacterial cells as Cu^2+^ via competitive interactions with other metal transport systems, particularly ABC-type metal transporters such as the Zn/Mn transporters (ZnuABC/MntABC) (Figure 1). The Zn transport system is well-characterized and relatively conserved across various bacterial taxa and has been extensively studied in *Synechocystis* sp. PCC 6803 [51,52]. Upon reaching the periplasm, Cu^2+^ can bind to the high-affinity periplasmic binding protein ZnuA, which subsequently delivers the ion to the inner membrane complex ZnuBC for translocation into the cytoplasm [51]. In both *Synechocystis* sp. PCC 6803 and *Escherichia coli*, Cu^2+^ has been shown to bind to the histidine-rich region of ZnuA, indicating at least one specific periplasmic interaction between Cu^2+^ and Zn-transporting proteins [51,53,54]. The transfer of Cu^2+^ from ZnuA to the membrane permease ZnuB is necessary for its translocation into the cytosol, while the ATPase subunit ZnuC supplies the energy required for active transport [55]. Furthermore, gene expression data from *Nostoc punctiforme* have shown that this operon is responsive to treatments with copper and other metals [56], suggesting that the Znu system may have a broader substrate range beyond zinc alone. The MntABC system functions via a binding and transport mechanism analogous to that of ZnuABC, as has been previously reported and studied in *Synechocystis* sp. PCC 6803 [57]. Although studies quantifying the interaction between Cu^2+^ and the MntABC system in cyanobacteria are currently lacking, copper uptake via this transporter has been demonstrated in *Staphylococcus aureus* and *Neisseria gonorrhoeae* [58,59].

CtaA is the only well-characterized protein known to exclusively mediate the import of Cu^2+^ from the periplasmic space across the plasma membrane into cyanobacterial cells [60]. It belongs to the P1B-type ATPase family, which includes specialized transporters responsible for the translocation of transition metal ions such as Cu^2+^, Zn^2+^, Cd^2+^, and other essential or potentially toxic heavy metals [61]. The structure of CtaA typically features six to eight membrane-spanning α-helices that form dedicated channels for metal ion transport. Additionally, it contains conserved residues—primarily histidines—in the N-terminal region that coordinate metal ions, functioning as sensors or facilitators of transport, along with ATP-binding domains that provide the energy required for active transport [62,63,64]. Although some studies propose that CtaA primarily functions in the efflux of Cu^2+^ into the periplasm [65], other evidence—including findings from cyanobacterial systems—suggests that CtaA also contributes to copper uptake, rather than serving solely in detoxification through export. Notably, mutants lacking CtaA typically exhibit increased resistance to excess copper, further supporting its role in copper homeostasis [52,66,67].

In addition to the CtaA protein, the FutABC iron transport system has also been implicated in copper translocation into the cytoplasm (Figure 1) [68]. This system plays a dual role in maintaining both iron and copper homeostasis in cyanobacterial cells. Primarily responsible for the uptake of iron from the periplasm into the cytoplasm, the FutABC transporter is embedded in the plasma membrane and actively contributes to regulating the intracellular Fe/Cu balance. The system comprises several components: FutA1 and FutA2, which bind iron in the periplasm; FutB, which forms the transmembrane channel; and FutC, an ATPase that provides energy for active transport [69]. In *Synechocystis* sp. PCC 6803, mutants lacking the *futA2* gene show copper accumulation in the periplasm, suggesting a role for FutA2 in copper handling [70]. Additionally, expression of *futB* is modulated in response to elevated copper levels in *Anabaena* sp. PCC 7120, further supporting the involvement of this system in copper homeostasis [71].

The cytoplasmic space is typically a highly reducing environment, where metal ions such as Cu^2+^ are readily reduced to the Cu^+^ form by redox-active molecules like glutathione and thioredoxins [72,73]. Once reduced, Cu^+^ binds to the metallochaperone Atx1, which facilitates its targeted delivery to the P1B-type ATPase complex PacS. PacS is responsible for transporting Cu^+^ into the thylakoid lumen, where it is essential for the proper functioning of copper-dependent proteins such as plastocyanin [11,60]. Atx1 contains a characteristic ferredoxin-like βαββαβ fold, featuring a conserved CXXC motif—comprising two cysteine residues separated by two variable amino acids—that mediates Cu^+^ binding. The β-α-β-β-α-β structure stabilizes the protein core and provides an optimal coordination environment for metal ion interaction, while the CXXC motif forms covalent bonds with Cu^+^, ensuring specific and efficient metal transfer [74,75,76]. Notably, studies in *Synechococcus* sp. PCC 7942 have shown that Atx1 interacts with the amino-terminal domains of both PacS and CtaA, suggesting that it functions as a copper shuttle between these two ATPases. Additionally, evidence indicates that Atx1 may positively regulate the function of Cu-dependent proteins within the thylakoid, further underscoring its critical role in intracellular copper trafficking and homeostasis [52].

A final factor that may impact Cu uptake by cyanobacterial cells is the presence of metallothioneins in the cytoplasm and their interaction with Cu ions. Metallothioneins are metal-binding peptides rich in cysteine and capable of binding a wide range of metal ions [77,78]. Although the classical role of these peptides is the sequestration of metal ions in general to prevent their excess from becoming harmful [79], it has been demonstrated in cyanobacteria that they may also participate in Cu uptake, probably in a “secondary” manner by sequestering free Cu in the cytoplasm and thus creating a favorable gradient for the entry of more Cu into the cell [80].

## 4. Response to Excess Cu

As elevated Cu levels are toxic to cyanobacterial cells, they have evolved active Cu extrusion systems whose expression is tightly regulated by intracellular Cu concentrations [34]. In *Synechocystis* sp. PCC 6803—the most extensively studied model—the accumulation of excess cytoplasmic Cu^2+^ activates a two-component regulatory system known as CopRS, which is part of the Resistance-Nodulation-Cell Division (RND) family. This system plays a central role in conferring Cu resistance by facilitating the export of toxic metal ions [34,60]. CopS, a membrane-associated histidine kinase (HK), serves as the sensor component of this system. It is localized in both the plasma and thylakoid membranes and binds Cu ions with high affinity [55]. Studies in *Escherichia coli* K-12 suggest that CopS possesses two transmembrane domains with peptide loops extending into the periplasm, allowing it to detect Cu throughout the intermembrane space [81]. Upon sensing elevated Cu levels, CopS is believed to autophosphorylate and subsequently transfer the phosphate group to its cognate response regulator, CopR. Phosphorylated CopR then functions as a transcriptional activator of Cu homeostasis genes [81,82,83]. This activation leads to the upregulation of two key operons involved in copper resistance: *copMRS* and *copBAC*, both of which are directly linked to Cu detoxification and export.

**Figure 2:**
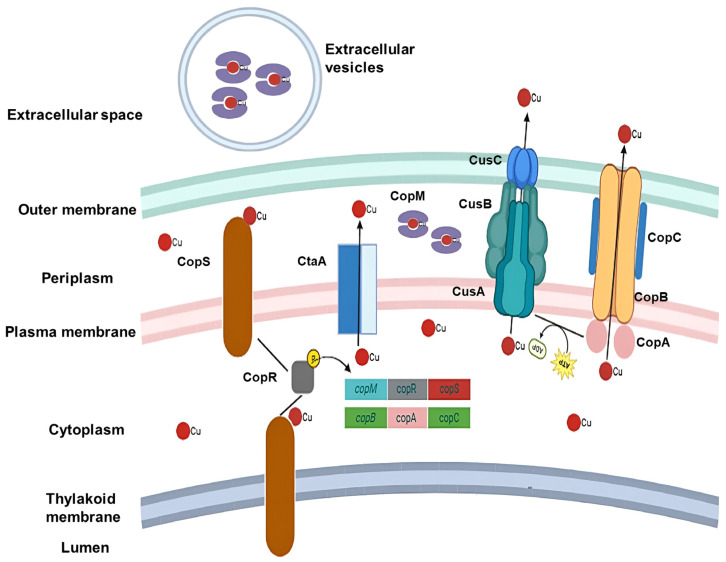
**Schematic representation of copper (Cu) ion response and efflux mechanisms in cyanobacteria.** This diagram illustrates the cellular processes involved in sensing and transporting Cu ions from the intracellular environment to the extracellular space. CopR/S (copper regulator/sensor proteins) are responsible for sensing Cu excess and increasing the transcription of related operons (copMRS and copBAC). The CopM (copper-binding protein), CopBAC, and CusABC (ABC efflux systems) complexes are directly responsible for the efflux of Cu ions. Arrows represent the Cu flux direction.

In *Synechocystis* sp. PCC 6803, the *copMRS* and *copBAC* operons are located on the native plasmid pSYSX, with the *copMRS* genes also found in the chromosomal genome [84]. Studies have shown that mutants lacking the *copR* gene exhibit reduced expression of these operons and increased sensitivity to copper stress [34]. As noted earlier, the transcriptional activator CopR enhances expression of both the *copRS* system and the *copM* gene [34]. CopM is a metallochaperone identified in the cytosolic, periplasmic, and extracellular compartments of cyanobacterial cells, capable of binding both Cu^2+^ and Cu^+^. It is believed to play a protective role against copper toxicity by immobilizing Cu ions, particularly in the extracellular environment [34]. Structural analysis revealed that CopM is a dimeric periplasmic protein composed of six α-helices arranged into a helical bundle [85]. This structure enables the protein to bind multiple Cu ions via its numerous methionine and histidine residues, which serve as coordination sites [86]. Although the mechanism of CopM export was previously unknown, recent findings have shown that CopM is present in extracellular vesicles isolated from *Synechocystis* sp. PCC 6803, where it interacts with Cu ions, suggesting a vesicle-mediated pathway for extracellular Cu sequestration (Figure 2) [87].

A higher intracellular concentration of Cu is required for CopR to activate the transcription of the *copBAC* operon compared to the concentration required to enhance copMRS expression [34]. The *copBAC* operon encodes a heavy metal efflux system, the CopBAC, classified as an HME-RND (heavy metal efflux/Resistance-Nodulation-Cell Division) type system [34]. This complex span both the plasma and outer membranes of cyanobacterial cells, extending across the periplasmic space [83].

Although detailed subcellular localization studies of each CopBAC subunit are limited, research on *Synechocystis* sp. PCC 6803 has identified CopB as a membrane fusion protein located in the periplasm, while CopA is a P-type ATPase RND associated with the inner membrane [84]. While not fully elucidated in cyanobacteria, CopC is recognized as a periplasmic Cu-binding protein with distinct binding sites for both Cu^+^ and Cu^2+^, likely involved in shuttling Cu to the efflux channel [88]. Comparative studies of Cu resistance mechanisms in other bacterial species have contributed to our understanding of such systems; however, the degree of homology between these bacterial and cyanobacterial proteins involved in Cu transport and resistance remains unclear and warrants further investigation [89,90,91,92,93].

**Figure 3:**
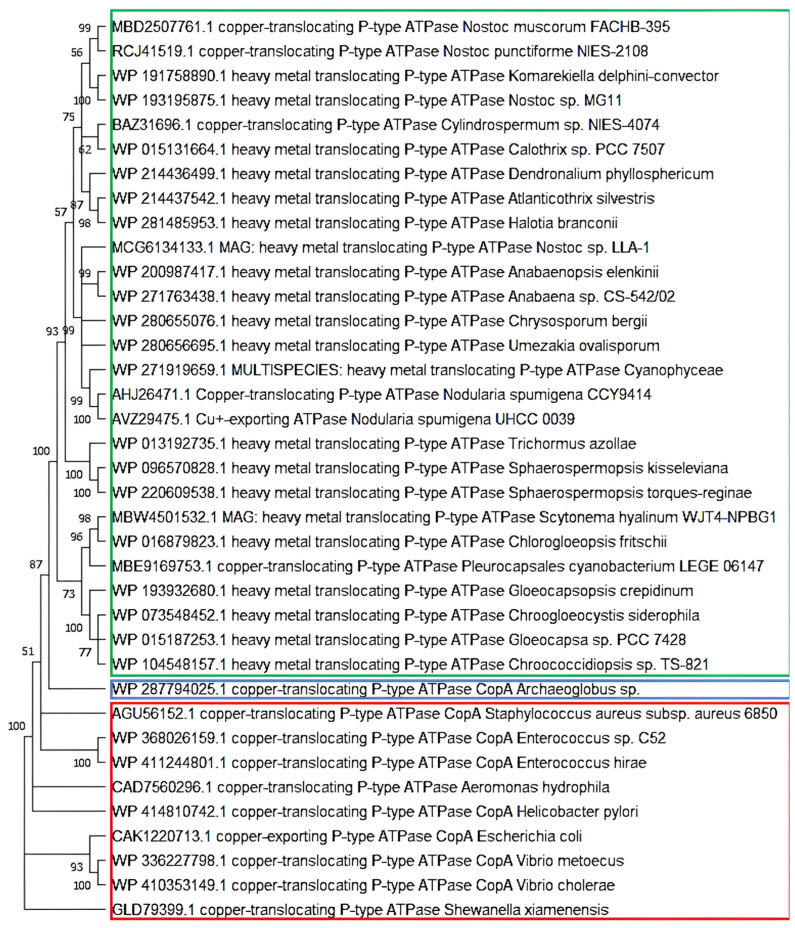
**Phylogeny of the functional domain P-type ATPase Cu exportation CopA protein in cyanobacteria.** Sequences with a single chain of approximately 750 amino acids and an identity of >65% among them. Archaea in blue, cyanobacteria in green, and other bacteria in red.

The components responsible for Cu response and efflux discussed in this section have been well characterized only in *Synechocystis* sp. PCC 6803 within the Cyanobacteria phylum, but it was possible to demonstrate that CopA (and probably CopB and CopC) is present throughout the phylum (Figure 3). Transcripts highly similar to *copM* have already been identified in *Anabaena* sp. PCC 7120 [94]. Other studies have shown that the CopR, CopS, and CopM components also exist in other bacterial phyla [86,95,96]. Therefore, while the presence of all these proteins requires experimental verification in other cyanobacterial strains, it is plausible that this system is conserved throughout the group.

Another system poorly studied in cyanobacteria but likely widespread in the phylum is the RND Cu and silver efflux system CusCBA (Figure 2), which is well described in the literature for *E. coli* [97]. The CusA protein is a proton-motive-force-dependent inner membrane RND efflux pump. CusC is a trimeric outer membrane porin that allows Cu to be directly expelled into the extracellular medium, while CusB is a hexameric membrane fusion protein capable of forming a structural bridge between CusA and CusC [98]. Studies in *Anabaena* sp. PCC 7120 have recorded the expression of genes homologous to *cusA* and *cusB* from *E. coli*, indicating that this system may also be present and play a role in Cu homeostasis in cyanobacterial cells [94,99,100].

Metallothioneins, already briefly discussed in the previous section, also play the classical role of Cu detoxification in cyanobacteria. Their binding to metal ions helps the cell reduce the excess of free ions. An example of a metallothionein in cyanobacteria is SmtA, which is composed of 56 amino acids. In *Synechococcus* PCC 7942, its expression is upregulated in response to excess Zn, Cd, and Cu, showing the highest responsiveness to elevated Zn levels [52].

## 5. Exopolysaccharides (EPS) Interactions

EPS function as a pivotal interface between cyanobacterial cells and their surrounding environment. While glucose predominates as the sugar-building block in EPS, other sugars, including rhamnose, xylose, arabinose, fucose, mannose, and uronic acids, have also been observed in certain cyanobacterial strains [101]. The classification of EPS is typically performed into two categories: capsular exopolysaccharides (CPS) and released exopolysaccharides (RPS). CPS have been observed to maintain a tight association with the cell surface, manifesting as sheaths, capsules, or slime layers. In contrast, RPS are known to be excreted into the extracellular space as free-floating polymers [102]. It has been demonstrated that both CPS and RPS exhibit distinct chemical and morphological characteristics. However, RPS has garnered greater industrial interest due to their larger production yields and greater independence in environmental interactions, facilitating metal biosorption [103,104,105,106,107].

It has been demonstrated that EPS are implicated in several essential biological functions, including the provision of structural integrity, facilitation of motility, and protection from various abiotic stresses [101]. The matrix of EPS is rich in charged functional groups, including carboxyl and hydroxyl side chains from deoxy-sugars, sulfate, and uronic acids [108]. This property enables EPS to interact with cationic species in solution. This interaction suggests that EPS may serve as a site for metal ion sequestration [109]. The interaction of EPS from various strains (whether bound to cells or isolated) with a wide range of environmental metal ions, including Cu, Pb, As, Cd, Cr, and Zn, has been shown to result in a significant reduction of these ions in the environment [110]. The overall negative charge of cyanobacterial EPS is particularly significant for the chelation of essential metal cations, which are present in trace amounts in the environment, as well as for mitigating direct contact with toxic heavy metals [103,108]. It has been demonstrated that an increase in Cu^2+^ concentrations can stimulate the production of EPS in *Nostoc spongiaeforme* [111] and substantial Cu^2+^ removal via EPS in *Cyanospira capsulata* and *Nostoc* PCC 7936 [112]. Furthermore, *Microcystis aeruginosa* exhibits a high affinity for Cu^2+^ ion adsorption, primarily through complexation [105].

Cu has been found to be one of the most efficiently adsorbed metals by cyanobacteria, surpassing other metal ions such as Ni and Zn [107]. Cu^2+^ ions have been observed to form complexes with anions such as CO_3_^2−^, OH^−^, and Cl^−^, as well as organic complexes with ligands including thiols, EPS, and humic substances [113]. The phenomenon of biosorption is characterized by the rapid, reversible, and passive binding of metal ions to functional groups present on the EPS surface, thereby protecting the cells themselves from the high concentration of metal ions [107]. However, it is important to note that the dynamics of the Cu-EPS interaction may be modified upon interaction with Cu^2+^ or other metal ions. For instance, alterations in pH have been demonstrated to influence the adsorption capacity and the specific binding sites between Cu^2+^ and EPS, as evidenced by studies involving EPS extracted from sludge in wastewater treatment [114].

## 6. Phylogeny and Structure of the CopA Protein

The structure of the CopA protein acting as P-type ATPase in Cu transport is probably the best studied among all the systems discussed in this review, as its primary structure is the most represented in online repositories, such as the NCBI (National Center for Biotechnology Information), and spans a diverse range of prokaryotic groups.

**Figure 4:**
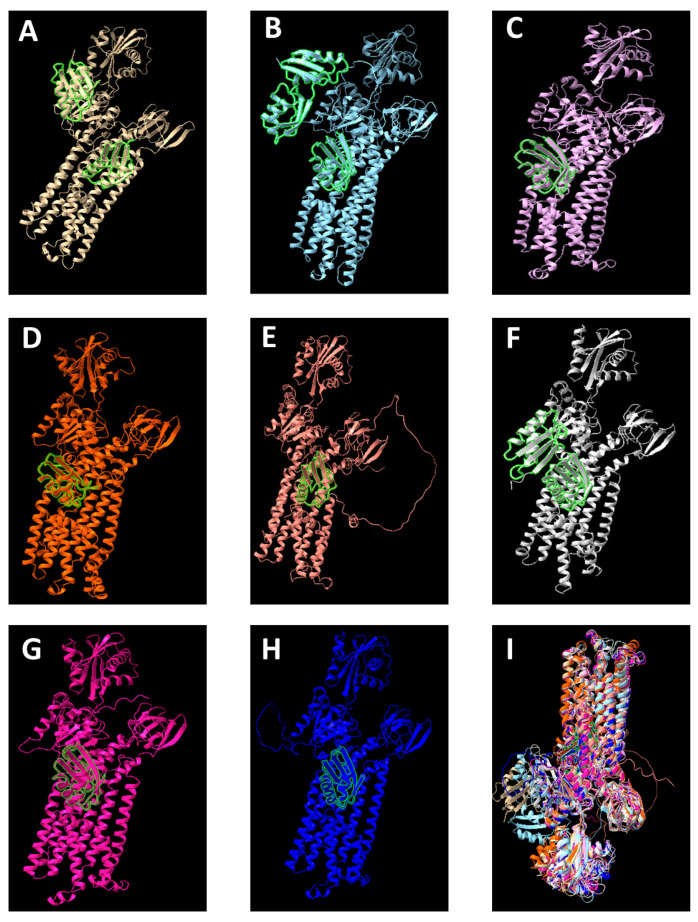
**3D model of the CopA protein in Cyanobacteria and other groups of prokaryotes constructed using ChimeraX.** (**A**) *Escherichia coli* O157:H7 (model obtained from Swiss-model; ~85.9 kDa); (**B**) *Vibrio cholerae* serotype O1 strain ATCC 39315 (model obtained from Swiss-model; ~96.4 KDa); (**C**) *Hydrococcus rivularis* NIES-593 (model obtained from Swiss-model; ~80.2 kDa); (**D**) *Gloeobacter violaceus* (model obtained from Swiss-model; ~77.2 kDa); (**E**) *Acidovorax* sp. 99 (model obtained from Swiss-model; ~86.3 kDa); (**F**) *Staphylococcus aureus* strain JH9 (model obtained from Swiss-model; ~86.7 kDa); (**G**) *Nodularia spumigena* UHCC0039 (model built using AlphaFold2 based on the sequence used for phylogeny; ~82.8 kDa); (**H**) *Nostoc muscorum* FACHB395 (model built using AlphaFold2 based on the sequence used for phylogeny; ~83.0 kDa); (**I**) Superposition of the proteins from a different perspective. The green markings highlight the **Heavy-Metal-Associated domain** of the proteins. The rest of the protein includes the **P-type ATPase Cu-like domain**.

The three-dimensional structure of the protein revealed that CopA is slightly smaller in the cyanobacterial phylum compared to other prokaryotic groups (Figure 4). This is because in cyanobacteria, CopA presents one Heavy-Metal-Associated domain and a P-type ATPase Cu-like domain, while in other organisms, CopA contains two Heavy-Metal-Associated domains and one P-type ATPase Cu-like domain. However, whether this is a unique and universal characteristic of the cyanobacterial phylum still requires further investigation.

The phylogeny of the P-type ATPase Cu-like domain of the CopA protein showed a clear division into three branches: cyanobacteria, other prokaryotes, and *Archaeoglobus* (Archaea) (Figure 3). This division is consistent with the phylogenetic history of these groups. Moreover, within the cyanobacterial branch, the phylogeny appeared to follow a pattern similar to that observed in trees based on the 16S rRNA molecular clock, indicating that this system is vertically distributed throughout the phylum as an ancient Cu homeostasis mechanism. This view is further supported by its well-conserved structure.

## 7. Conclusions

This review underscores the critical role of copper (Cu) homeostasis in cyanobacterial physiology, with a focus on the molecular components that mediate Cu uptake—from the extracellular environment to the thylakoid lumen—and Cu efflux under conditions of excess. We highlight key proteins involved in these pathways, their subcellular localization, and the genes regulated in response to both Cu deficiency and toxicity. Particular attention is given to the potential involvement of extracellular polymeric substances (EPS) in Cu binding and detoxification. These insights provide a more integrated understanding of the complex Cu transport network in cyanobacteria, offering a foundation for future applications in bioremediation and the development of Cu-based strategies for controlling harmful cyanobacterial blooms in aquatic ecosystems.

Additionally, we identified key gaps in current knowledge, including the need for further investigation into Cu limitation and the effects of sub-toxic concentrations, as well as the functional characterization of poorly studied systems such as CusABC. Our phylogenetic and structural analysis of the CopA protein reveals notable findings, including the widespread presence of the CopBAC system across the phylum and the unique feature of cyanobacterial CopA harboring a single functional metal-binding domain—contrasting with the dual-domain structure observed in many other prokaryotes. Importantly, here, we compiled data from a wide range of cyanobacterial strains beyond the commonly used model organisms, aiming to offer a more comprehensive overview of Cu homeostasis across the phylum.

## Data Availability

The data that support the findings of this study are available from the corresponding author upon reasonable request.

## Abbreviations

The following abbreviations are used in this manuscript:

Cd	cadmium
CPS	capsular exopolysaccharides
Cr	chromium
Cu	copper
Cyt c6	cytochrome c6
EPS	exopolysaccharides
Fe-S	iron-sulfur
Hg	mercury
HK	histidine kinase
LC50	lethal concentration 50
Mn	manganese
Ni	nickel
NCBI	National Center for Biotechnology Information
PC	plastocyanin
Pb	lead
PSI	photosystem I
PSII	photosystem II
RND	Nodulation-Cell Division
ROS	reactive oxygen species
RPS	released exopolysaccharides
SLH	S-layer Homologous
SOD	superoxide dismutase
Zn	zinc
